# Effect of a family and interdisciplinary intervention to prevent T2D: randomized clinical trial

**DOI:** 10.1186/s12889-020-8203-1

**Published:** 2020-01-22

**Authors:** Katya Vargas-Ortiz, Georgina Lira-Mendiola, Claudia M. Gómez-Navarro, Katya Padilla-Estrada, Fabiola Angulo-Romero, José M. Hernández-Márquez, Ana K. Villa-Martínez, Jessica N. González-Mena, Maciste H. Macías-Cervantes, Maria de Lourdes Reyes-Escogido, Rodolfo Guardado-Mendoza

**Affiliations:** 1 Department of Medical Sciences, University of Guanajuato, Campus León, Guanajuato, Mexico; 2 Metabolic Research Laboratory, Department of Medicine and Nutrition, University of Guanajuato, Campus León, Blvd. Puente Milenio No. 1001 Fracción del Predio San Carlos C.P. 37670; León, Guanajuato, Mexico

**Keywords:** Family support, Interdisciplinary, Intervention, Prediabetes, Prevention

## Abstract

**Background:**

Lifestyle changes can reduce the risk of T2D; however, no study has evaluated the effect of a lifestyle intervention involving patients´ family. The aim of this study was to compare the impact of an interdisciplinary family (FI) Vs individual intervention (II) on glucose metabolism, insulin resistance (IR), pancreatic β-cell function and cardiovascular risk markers in patients with prediabetes, as well as to measure the impact on their families’ metabolic risk.

**Methods:**

Randomized Clinical Trial (RCT) to compare the impact of FI and II on IR and pancreatic β-cell function in subjects with prediabetes. There were 122 subjects with prediabetes (and 101 family members) randomized to FI or II. Data were collected in 2015–2016 and analyzed in 2017–2018. FI group had the support of their family members, who also received personalized diet and exercise recommendations; patients and their family members attended monthly a lifestyle enhancement program. II group received personalized diet and exercise recommendations. The follow-up was for 12 months. Glucose, IR, pancreatic β-cell function and secondary outcomes (body composition and lipid profile) were assessed at baseline, 6 and 12 months.

**Results:**

FI group improved area under the glucose curve (AUC) (from 18,597 ± 2611 to 17,237 ± 2792, *p* = 0.004) and the Matsuda index (from 3.5 ± 2.3 to 4.7 ± 3.5, *p* = 0.05) at 12 months. II group improved Disposition Index (from 1.5 ± 0.4 to 1.9 ± 0.73, *p* < .0001) at 12 months.

The improvements achieved in weight and lipids at 6 months, were lost in II group at 12 moths, whereas in FI persisted. Adherence up to 12 months was not different between the study groups (FI 56% Vs II 60%).

**Conclusions:**

FI intervention was more effective by improving glucose AUC, insulin sensitivity and lipid profile, besides that, metabolic risk in family members of the FI group was maintained, while the risk of II group was increased.

**Trial registration:**

This study was retrospectively registered at clinicaltrials.gov on December 15, 2015 (NTC026365646).

## Background

Prediabetes is an abnormality in glucose levels that occurs before type 2 diabetes (T2D), and it is diagnosed when fasting glucose levels (FPG) are between 100 and 125 mg/dl, when 2 h plasma glucose values after a 75 g oral glucose tolerance test (OGTT) are between 140 and 199 mg/dl, or HbA1c is between 5.7–6.4% [1]. Annual incidence of T2D in subjects with prediabetes is higher (4–10%) than in subjects with normoglycemia(< 0.5%) [2, 3]. Overweight/obesity and sedentary lifestyle are directly related to prediabetes, and that is why lifestyle programs may be useful to prevent T2D.

Lifestyle interventions have shown to be one of the best strategies to reduce the risk of T2D in subjects with prediabetes [4–6]. In the Diabetes Prevention Program (DPP) study, in addition to lifestyle intervention group, there was a group treated with metformin, and it was observed that lifestyle was more effective to reduce the incidence of T2D [7], although this effect was progressively reduced with time [8], perhaps influenced by the low adherence to lifestyle and the progressive nature of the disease [9].

Most of the previous lifestyle intervention studies in prediabetes have not considered the importance of family support. In patients with T2D, family support has been reported to have an impact on the disease prognosis [10]. In cross-sectional studies, the family support on T2D management was associated with glucose levels < 140 mg/dl [11] and improvements on metabolic outcomes [12]. The family, as a universal social system, it is the first contact with the patient and may have different influences on him. A lack or insufficient support from the nearest nucleus of the patient can predispose to metabolic decontrol by limiting or obstructing the adequate control and treatment of the disease. In general population there is a low adherence to treatment prescriptions and changes in lifestyle are extremely difficult, sometimes due to lack of family support, which affects optimal control in patients with T2D [13]. Thus, family support could play a key role in metabolic control of the disease by providing a favorable environment that reduces stress and improves compliance in patients, and equally important, family support could achieve a healthier status in family members.

The benefits of lifestyle interventions in patients with abnormalities in glucose metabolism and the importance of family support in the management of T2D have been reported in previous studies, however, no lifestyle intervention study has involved the family as a support in the management of prediabetes. Insulin resistance and pancreatic β-cell function are key markers of glucose metabolism abnormalities in prediabetes and in the progression from prediabetes to T2D [14–16] and patients with prediabetes have already a higher cardiovascular risk [17, 18].

The goal of this work was to compare the impact of an interdisciplinary family intervention (FI) vs individual intervention (II) on glucose metabolism, insulin resistance (IR), pancreatic β-cell function and cardiovascular risk markers in patients with prediabetes, as well as to measure the impact on their families’ metabolic risk.

## Methods

The study protocol was approved by the Research Council at The University of Guanajuato and it was retrospectively recorded in the National Clinical Trials Registry on December 15, 2015 (NTC026365646). A written informed consent was signed by all participants.

### Subjects

Eligibility criteria were: age of 18–60 years and diagnosis of prediabetes: FPG between 100 and 125 mg/dl and/or 2 h OGTT glucose between 140 and 199 mg/dl [19]. Patients were not included if they were taking medications known to affect glucose metabolism and excluded if they become pregnant during the study. The participants were Mexican residents from Guanajuato state. Data were collected in 2015–2016 and analyzed in 2017–2018.

### Study design

This was a parallel RCT of 6 months with an open follow-up to 12 months; patients were recruited from a metabolic screening program performed in general population as part of the University Cohort Project CARE-in-DEEP Study (Cardiometabolic Risk Evaluation and Interdisciplinary Diabetes Education and Early Prevention). Initial screening was conducted using a general survey of lifestyle information. All participants (patients and family members) attended to the Metabolic Research Laboratory at the University of Guanajuato.

Participants with prediabetes were randomly assigned to one of two interventions, by a simple randomization electronic method performed by a Nutritionist not involved in the study. Personal who evaluated outcomes were also blinded to the participant’s treatment group.

The duration of intervention was 6 months with an open follow-up period up to 12 months. Patients in both groups received also metformin at a starting dose of 850 mg once daily and progressively increasing to 850 mg twice daily during the next month.

### Baseline evaluation

#### Anthropometrical measurements

Weight was measured while participants were barefoot and wearing minimal clothing. Height was obtained while the participants were standing barefoot with their shoulders in a normal position. Body mass index (BMI) (kg/m^2^) was obtained from standardized measurements of weight and height and was computed as a ratio of weight (kg): height squared (m^2^). Waist circumference was measured at the high point of the iliac crest at the end of normal expiration to the nearest 0.1 cm. Body composition (body fat percentage and visceral index) was assessed with electrical bioimpedance through a Tanita Scale SC-240. All measurements were performed by personal trained to use standardized procedures and reproducibility was evaluated, resulting in concordance coefficients between 0.88 and 0.94.

#### Nutritional and physical activity evaluation

A semi-quantitative food frequency questionnaire previously validated [20], was applied to evaluate dietary intake. This questionnaire included data regarding the consumption of 116 food items and participants reported their frequency of consumption for each specific food during the previous year. Total energy intake was computed by summing the energy intakes from all foods.

The Physical Activity (PA) level of participants was assessed using the long version of self-administered International Physical Activity Questionnaire that was verified when the patients assisted for the metabolic evaluation. The scores are expressed in METs -minutes/week.

#### Clinical and metabolic evaluation

Resting blood pressure was measured twice by means of the auscultatory method using a sphygmomanometer in the seated position (HEM-714; Omron Healthcare, Inc., USA). The average of two blood pressure measures was used in the analysis [21].

Oral glucose tolerance test (OGTT): All subjects were admitted to the Metabolic Research Laboratory the day of the study between 7 and 8 AM, and a catheter was placed into an antecubital vein for all blood withdrawal. Subjects will not be allowed to eat or drink anything after 10 PM on the night before, until the study is completed. After the intravenous catheter was placed and the first blood sample was drawn, the patients ingested 75 g of glucose. Plasma samples for glucose measurement were drawn at 0 min and every 30 min thereafter for two hours. Glucose and lipid levels were measured by a colorimetric method (Kodak Ektachem DT60II). Insulin was measured by chemiluminescence (IMMULITE 1000) and different indices of IR, insulin secretion and β-cell function (disposition and oral disposition indexes), were calculated. Glycated hemoglobin (HbA1c) was measured according to the American Diabetes Association (ADA) recommendations using an HPLC method.

#### Calculations

The area under the curve (AUC) for glucose and insulin during OGTT were calculated according to the trapezoidal rule. Insulin secretion was calculated dividing the AUCinsulin_0_120min_ by the AUCglucose_0_120min_ during the OGTT. Disposition index (the insulin secretion/insulin resistance (IS/IR) index) during OGTT was calculated as (AUCinsulin_0_120min_ / AUCglucose_0_120min_)*Matsuda index [22]; oral disposition index was calculated by (insulin change from 0 to 30 min / glucose change from 0 to 30 min) / (1/fasting insulin). Insulin sensitivity during OGTT was calculated from the Matsuda index [23], and at fasting with the homeostasis model assessment (HOMA-IR).

### Interventions

#### Individual intervention

In addition to metformin 850 mg twice daily, patients were prescribed a diet consisting of 50–60% carbohydrates, 15–20% proteins and less than 30% total fat. In case of overweight or obesity the patients received detailed nutrition advice to achieve a 5–7% reduction in body weight and a moderate caloric restriction to lose weight was prescribe (250 to 500 kcal less than ingestion daily average calculated in the dietary regimen). Dietary recommendations were customized considering the 3-day food records; reductions in caloric intake were gradually through the study, adjusting the diet every two months. If patient had a sedentary lifestyle, it was advised to start with 45 min/week of mild to moderate exercise (the chosen activity was according to patient preference), a daily frequency was recommended or at least every third day. Patients were counseled to increase the time or intensity of exercise every two weeks until reaching 150 min/week of moderate activity or 75 min/week of intense activity. If the patient was already physically active, it was recommended to continue like this and vary its exercise routines.

#### Interdisciplinary family intervention

Patients in this group had also the intervention mentioned above, and their family members also received a personalized nutritional counseling and diet. In addition, patients and their family members attended a monthly lifestyle enhancement program. This program consisted on 6 monthly group sessions with duration of approximately 1 h with an interdisciplinary approach to address issues about nutrition, physical activity and stress management. Nutrition topics were management of the meal plan, plate of good food, understanding and comparison of labeling of food products, eating with attention and healthy lunches; the topics about physical activity were concerning safety about physical activity, benefits of exercise, risks of sedentary lifestyle and detraining, progression of routines, types of exercise and physical training at home; covered topics to face stress management were commitment, relation between body, mind and emotions, evaluation of commitments and relaxing exercises. Sessions were prepared and conducted by an interdisciplinary group (nutritionist, general physician, physical activity specialist, psychologist, and endocrinologist). Additional information file shows this in more detail [see Additional file 1].

Patients in both intervention groups had monthly follow-up for 12 months. At each follow-up visit the body composition, fasting glucose, adherence to pharmacotherapy (by pill count), diet and exercise (by 3-day food records and the International Physical Activity Questionnaire [IPAQ]) were revised. The pertinent recommendations were given to each patient in order to achieve better adherence in all aspects of the intervention.

At 6 months, baseline evaluation was repeated in patients, while only body composition, fasting glucose and lipid profile were measured in their family members. After 12 months baseline evaluation was repeated in patients and their family members.

### Main outcome

Primary outcomes: glucose, IR, and pancreatic β-cell function at 6 and 12 months. Secondary outcomes were anthropometric and lipid profile changes.

Patients diagnosed with T2D during the study follow-up, based on repeated measurements of FPG, 2 h glucose, or HbA1c, continued their specific treatment with their family physicians.

### Statistical analysis and sample size

Sample size calculation was performed for comparison of two proportions. We expect that family and interdisciplinary intervention improves individual and family profiles of glucose, insulin secretion and insulin resistance in at least 60% of patients, while individualized intervention improves them in approximately 30% of patients [5–7, 24]; therefore, the study was designed to detect a minimum difference of 30% between groups; with a type I (alpha) error of 0.05 and a sample power of 80%, the minimum sample size would be 41 patients per group, that including a 20% of expected losses, it would be 50 patients per group. Comparisons between groups were performed using an independent t test and intra-groups differences were assessed by ANOVA for repeated measurements, using a per protocol analysis. A *p* value < 0.05 was considered as statistically significant. SPSS, version 21 was used to perform statistical analysis.

## Results

**Fig. 1:**
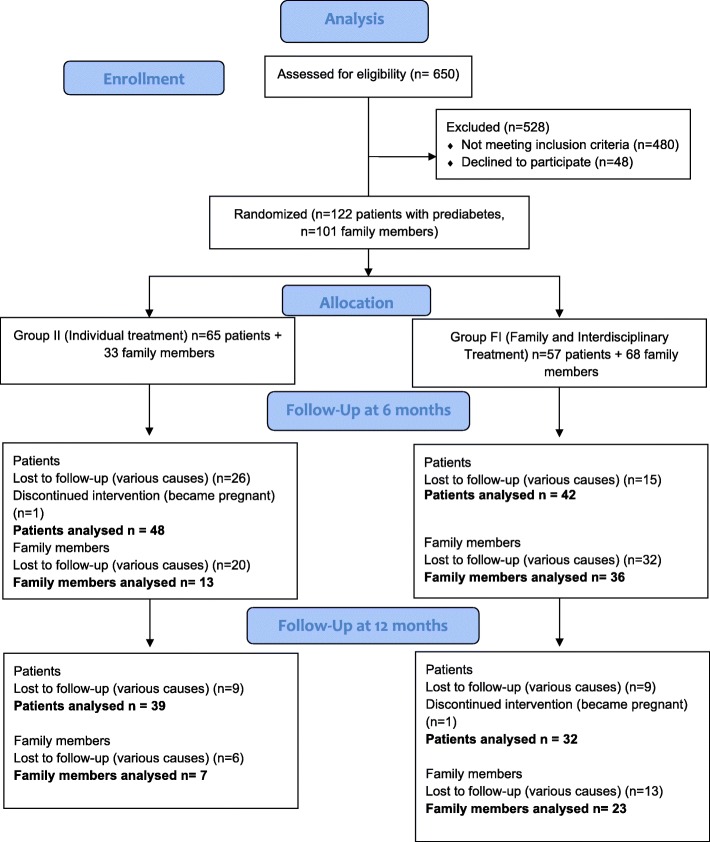
Flow of participants through the trial

As we can see in Fig. 1, 98 participants were included into the II group (65 patients with prediabetes and 33 family members with normoglycemia), and 125 participants into the FI group (57 patients with prediabetes and 68 family members with normoglycemia). Sixty-one participants in the II group achieved the follow-up at 6 months, and only 46 returned for the evaluation at 12 months. On the other hand, in the FI group 78 participants achieved the follow-up at 6 months and 55 returned for the 12 months evaluation. Although some patients left the intervention due to specific causes such as pregnancy (one patient of the each group), change of address (one patient of the II group), new employment (one patient oh the FI group) and no localization (7 patients of the II group and 5 of the FI group), the highest proportion of drop-outs was due to lack of interest from the patients. This shows the low adherence to prevention programs in the general population, and the lack of availability to spend time on health care when participants do not feel themselves sick.

It is important to note that the patients who finished 12 months of follow-up and those who did not, had similar glucose metabolism, anthropometric and pathophysiological characteristics, they were different only in age (48 ± 11 vs 41 ± 13 years, *p* = 0.001), total cholesterol (196.7 ± 4.2 vs 179.6 ± 4.2 mg/dl, *p* = 0.005) and LDL cholesterol (121.5 ± 4.1 vs 108.8 ± 4.0 mg/dl, *p* = 0.03). There were no significant differences between family members who finished and did not finish the 12 months follow-up.

Baseline characteristics were similar between the study groups. Average BMI was in the obesity range (> 30 kg/m^2^, Table 1), age was 46 ± 12 and 44 ± 13 years for II and FI group, respectively, and basic socio-demographic characteristics as TD2 family history were similar between groups (Table 2). Adherence to metformin in both groups was greater than 80%. Taking into consideration the IPAQ guidelines, both groups had a moderate physical activity classification and their caloric intake was 1860 ± 1201 and 2007 ± 717 kcal (II and FI, respectively) without significant differences between them.

**Table 1:** Clinical and biochemical characteristics between the study groups before and after intervention

Anthropometric Characteristics	Individual Group (*n* = 65)			Familiar Group (*n* = 57)		
	Basal (*n* = 65)	Month 6 (*n* = 48)	Month 12 (*n* = 39)	Basal (*n* = 57)	Month 6 (*n* = 42)	Month 12 (*n* = 32)
Weight (kg)	81.8 ± 17.0	**75.4 ± 17.5**^**B**^	**76.7 ± 19.8** ^**C**^	82.1 ± 18.0	**78.5 ± 19.0** ^**E**^	**76.2 ± 15.0** ^**F**^
BMI (kg/m^2^)	30.2 ± 5.3	**28.3 ± 5.5**^**B**^	**28.9 ± 6.5** ^**C**^	30.3 ± 5.8	**29.2 ± 6.0** ^**E**^	**28.6 ± 4.6**^**F**^
WC (cm)	95.3 ± 12.0	**91.5 ± 12.4** ^**B**^	93.5 ± 13.5	96.2 ± 14.0	92.8 ± 17.4	92.8 ± 10.5
Body fat (%)	34.8 ± 8.3	**32.7 ± 8.8** ^**B**^	35.6 ± 7.6	34.8 ± 8.3	**33.6 ± 9.0** ^**E**^	34.5 ± 9.0
Visceral Index (AU)	11.0 ± 4.8	**9.8 ± 4.6**^**B**^	11.3 ± 4.9	11.3 ± 4.6	**11.0 ± 4.7** ^**E**^	**10.6 ±** **4.3** ^**F**^
SBP (mm Hg)	123 ± 16	119 ± 16.7	121 ± 14	116 ± 11	113 ± 12	122 ± 15
Biochemistry Characteristics						
Glucose (mg/dl) 0 min	101 ± 10	**97 ± 10**^**B**^	**94 ± 10**^**C**^	104 ± 9	**97 ± 11** ^**E**^	**98 ± 8** ^**F**^
Glucose (mg/dl) 120 min	146 ± 23	**131 ± 45**^**B**^	**127 ± 33**^**C**^	149 ± 27	**135 ± 35**^**E**^	142 ± 33
HbA1c (%)	5.3 ± 0.5	5.4 ± 0.3	5.4 ± 0.3	5.1 ± 0.7	**5.5 ± 0.4**^**E**^	**5.4 ± 0.3** ^**F**^
AUCgluc_OGTT (mg/dl/120 min)	18,398 ± 2403	17,749 ± 3965	17,204 ± 3429	18,597 ± 2611	**17,637 ± 3849**^**E**^	**17,237 ± 2792**^**F**^
Tg (mg/dl)	159 ± 79	135 ± 58	148 ± 58	192 ± 90	167 ± 79	210 ± 164
Total Cholesterol (mg/dl)	181 ± 34	177 ± 35^**∆H**^	183 ± 35^**∆I**^	197 ± 40	**186 ± 38**^**E, ∆H**^	**186 ± 35**^**F∆I**^
HDL (mg/dl)	40 ± 11	44 ± 13	39 ± 14	42 ± 11	44 ± 16	44 ± 12
LDL (mg/dl)	112 ± 33	108 ± 27	118 ± 32^**∆I**^	121 ± 36	**108 ± 33**^**E**^	**106 ± 38**^**F, ∆I**^
Markers of insulin resistance and pancreatic β-cell function						
Matsuda Index	3.9 ± 1.9	**4.8 ± 2.6**^**B**^	**4.9 ± 3.0**^**C**^	3.5 ± 2.3	**4.9 ± 3.5**^**E**^	**4.7 ± 3.5**^**F**^
HOMA IR	2.9 ± 2.0	**2.4 ± 1.9**^**B**^	**2.4 ± 1.7**^**C**^	3.5 ± 2.0	2.9 ± 3.4	3.1 ± 2.7
Insulin secretion	0.45 ± 0.20	0.44 ± 0.22	0.49 ± 0.25^**∆I**^	0.58 ± 0.38	0.48 ± 0.26	0.50 ± 0.27^**∆I**^
Disposition Index	1.5 ± 0.4	**1.8 ± 0.7**^**B**^	**1.9 ± 0.7**^**C∆I**^	1.5 ± 0.5	**1.9 ± 0.9**^**E**^	1.7 ± 0.5^**∆I**^
Oral Disposition Index	0.11 ± 0.12	0.15 ± 0.18	0.08 ± 0.14	0.13 ± 0.13	0.11 ± 0.12	0.11 ± 0.06

Data presented as means ± standard deviation. A = *p* < 0.05 between groups at the corresponding time; B = *p* < 0.05 basal vs month 6 individual group; C = *p* < 0.05 basal vs month 12 individual group; D = *p* < 0.05 month 6 vs month 12 individual group; E = *p* < 0.05 basal vs month 6 familiar group; F = *p* < 0.05 basal vs month 12 familiar group; G = *p* < 0.05 month 6 vs month 12 familiar group; ∆H = *p* < 0.05 for comparison of change between groups from 0 to 6 months; ∆I = *p* < 0.05 for comparison of change between groups from 0 to 12 months. AU, arbitrary units; AUCgluc_OGTT, area under the glucose curve during oral glucose tolerance test; AUCIns, area under the insulin; BMI, body mass index; HbA1c, Glycated hemoglobin; HDL, high density lipoprotein; HOMA IR, homeostatic model assessment; IR, insulin resistance; LDL, low density lipoprotein; SBP, systolic pression blood; Tg, triglycerides; WC, waist circumference

**Table 2:** Socio-demographic characteristics between the study groups

Socio-demographic Characteristics	Prediabetic patients			Family Members		
	Individual Group (*n* = 65)	Familiar Group (*n* = 57)	P	Individual Group (*n* = 33)	Familiar Group (*n* = 68)	p
Age (years)	46 ± 12	44 ± 13	**NS**	32 ± 9	33 ± 13	**NS**
Female, n (%)	33 (50.7)	32 (56.1)	**NS**	22 (66.6)	42 (61.7)	**NS**
Education level						
Primary school, n (%)	8 (12.3)	10 (17.5)	**NS**	0 (0.0)	2 (2.9)	**NS**
High school, n (%)	19 (29.2)	20 (35.1)	**NS**	10 (30.3)	36 (52.9)	**NS**
University, n (%)	38 (58.5)	27 (47.4)	**NS**	23 (69.7)	30 (44.2)	**NS**
Civil status						
Married, n (%)	42 (64.6)	44 (77.2)	**NS**	19 (57.6)	28 (41.2)	**NS**
Single, n (%)	11 (16.9)	5 (8.8)	**NS**	14 (42.4)	28 (41.2)	**NS**
Free union, n (%)	2 (3.1)	4 (7.0)	**NS**	0 (0.0)	7 (10.3)	**NS**
Divorced, n (%)	6 (9.2)	1 (1.7)	**NS**	0 (0.0)	2 (2.9)	**NS**
Separated, n (%)	3 (4.6)	2 (3.6)	**NS**	0 (0.0)	2 (2.9)	**NS**
Widow, n (%)	1 (1.5)	1 (1.7)	**NS**	0 (0.0)	1 (1.4)	**NS**
With T2D Family history, n (%)	52 (80.0)	51 (89.5)	**NS**	26 (78.8)	54 (79.4)	**NS**

Data presented in number and percentage. *NS* not significant

Patient’s family members had an average BMI in the overweight range (BMI > 26 kg/m^2^), they were 32 ± 9 and 33 ± 13 years old (II and FI, respectively) (Tables 2–3). Family members of both groups had similar socio-demographics characteristics, T2D family history (Table 2) and had a moderate PA classification, their caloric intake was 1759 ± 1084 and 1957 ± 872 kcal (II and FI, respectively) without significant differences between them.

**Table 3:** Family members characteristics by intervention groups through the study

Anthropometric Characteristics	Individual Group (*n* = 33)			Familiar Group (*n* = 68)		
	Basal *(n = 33)*	Month 6 *(n = 13)*	Month 12 *(n = 7)*	Basal *(n = 68)*	Month 6 *(n = 36)*	Month 12 *(n = 23)*
Weight (kg)	72.0 ± 17.0	66.4 ± 16.0	**75.0 ± 16.0**^**C**^	68.6 ± 17.0	68.5 ± 16.0	65.4 ± 16.8
BMI (kg/m^2^)	26.3 ± 5.4	25.0 ± 5.4	**28.4 ± 5.2**^**C**^	25.9 ± 5.2	26.3 ± 5.3	25.5 ± 5.3
WC (cm)	86.0 ± 12.6	**82.6 ± 11.8**^**B**^	88.7 ± 11.2	83.6 ± 17.2	83.9 ± 11.8	78.8 ± 17.3
Body fat (%)	29.0 ± 11.0	28.2 ± 11.7	**30.8 ± 13.0**^**C**^	28.3 ± 9.0	28.6 ± 10.1	28.9 ± 10.1
Visceral Index (AU)	5.5 ± 4.0	4.7 ± 3.3	5.4 ± 3.9	6.3 ± 4.6	6.4 ± 4.6	5.0 ± 3.7
SBP (mm Hg)	108 ± 10	109 ± 10	111 ± 7	108 ± 14	110 ± 10	111 ± 13
Biochemistry Characteristics						
Glucose (mg/dl) 0 min	89 ± 8.0	91 ± 10	88 ± 7	92 ± 9.0	90 ± 10	93 ± 10
Glucose (mg/dl) 120 min	106 ± 22	Not done	104 ± 29	102 ± 19	Not done	100 ± 22
AUCgluc_OGTT (mg/dl/min)	14,262 ± 2342	Not done	14,556 ± 2410	13,691 ± 2534	Not done	13,966 ± 2679
Tg (mg/dl)	103 ± 38	118 ± 36	108 ± 40	123 ± 58	128 ± 65	112 ± 62
Total Cholesterol (mg/dl)	167 ± 29	161 ± 30	180 ± 39^**∆I**^	174 ± 38	167 ± 38	159 ± 30^**∆I**^
HDL (mg/dl)	45 ± 11	50 ± 14	46 ± 12	44 ± 12	43 ± 11	50 ± 11
LDL (mg/dl)	102 ± 28	87 ± 28	**112 ± 38**^**C, ∆I**^	104 ± 32	**102 ± 30**^**E**^	**90 ± 29**^**F, ∆I**^
Markers of insulin resistance and B cell function						
Matsuda Index	6.3 ± 3.9	Not done	4.6 ± 3.5	5.2 ± 3.2	Not done	5.5 ± 2.6
HOMA IR	1.8 ± 0.9	Not done	2.4 ± 1.0	2.3 ± 1.4	Not done	2.4 ± 1.8
Insulin secretion	**0.47 ± 0.25** ^**A**^	Not done	0.75 ± 0.38	**0.66 ± 0.35** ^**A**^	Not done	0.60 ± 0.33
Disposition Index	2.38 ± 0.75	Not done	2.64 ± 0.55	2.68 ± 0.86	Not done	2.62 ± 0.87

Data presented as means ± standard deviation. A = *p* < 0.05 for comparison between groups at the corresponding time; B = *p* < 0.05 basal vs month 6 individual group; C = *p* < 0.05 basal vs month 12 individual group; D = *p* < 0.05 month 6 vs month 12 individual group; E = *p* < 0.05 basal vs month 6 familiar group; F = *p* < 0.05 basal vs month 12 familiar group; G = *p* < 0.05 month 6 vs month 12 familiar group; ∆H = *p* < 0.05 for comparison of change between groups from 0 to 6 months; ∆I = *p* < 0.05 for comparison of change between groups from 0 to 12 months. AU, arbitrary units; AUCgluc_OGTT, area under the glucose curve during oral glucose tolerance test; AUCIns, area under the insulin curve; BMI, body mass index; HbA1c, Glycated hemoglobin; HDL, high density lipoprotein; HOMA IR, homeostatic model assessment; IR, insulin resistance; LDL, low density lipoprotein; SBP, systolic pression blood; Tg, triglycerides; WC, waist circumference

### Six months follow-up

At 6 months of follow-up all anthropometric characteristics improved significantly in both groups, with no differences between them (Table 1). Caloric intake and physical activity were not different between groups. Nevertheless, calorie intake at 6 months of follow-up decreased in II group by 3%, while in the FI group decreased by 7%. Changes in macronutrients intake were the following: In II group fat intake decreased 24%, carbohydrates and protein intake increased 15 and 11% respectively. In FI group fat, carbohydrates and protein intake decreased 8, 6 and 4% respectively. On the other hand, the amount of PA at 6 months decreased by 7% in the II group, and it increased by 12% in the FI group.

Both groups showed significant improvements in glucose values, in addition, FI group also improved the area under the glucose curve, LDL and total cholesterol, being these improvements in lipid levels significantly better in the FI group in comparison to the II group (Table 1, *p* = 0.04).

Both groups improved IR and pancreatic β cell function at 6 months. HOMA-IR was improved in both groups although this difference was statistically significant only in II group (− 0.50 CI 95% -0.77 to − 0.06). Insulin sensitivity (IS) (Matsuda Index) was significantly improved mostly in the FI group (1.39 CI 95% 0.30–2.48); in addition, both groups improved significantly pancreatic β-cell (disposition index), FI group increased 0.40 (CI 95% 0.09–0.64) and the II group increased 0.30 (CI 95% 0.10–0.47) (Table 1).

Family members of both groups did not show significant changes at 6 months of follow-up, there was only a significant reduction of waist circumference in II and in LDL levels in FI group (Table 3).

### Twelve months follow-up

Only 39 and 32 patients, and 7 and 23 family members (II and FI respectively), completed the 12 months follow-up. At this time, several improvements achieved by patients at 6 months were lost in the II group, whereas in FI group these improvements were maintained or even improved; visceral fat index in II group at 0, 6 and 12 months was 11.0 ± 4.8, 9.8 ± 4.6 and 11.3 ± 4.9 respectively; while in FI group it was 11.3 ± 4.6, 11.0 ± 4.7 and 10.6 ± 4.3 respectively. However, there were not statistically significant differences in the changes in anthropometric variables between groups (Table 1).

Regarding glucose metabolism, there were not significant differences at 12 months between groups. On the other hand, in II group the lipid profile worsened not only with respect to the 6 months of follow-up, also with respect to baseline measurement (without significant difference) (Table 1); while the significant improvements in total cholesterol (from 197 ± 40 to 186 ± 35 mg/dl) and LDL (from 121 ± 36 to 106 ± 38 mg/dl) in the FI group persisted up to 12 months; thus, the change from 0 to 12 months in total cholesterol and LDL were significantly better in the FI group vs II group (total cholesterol, *p* = 0.007 and LDL, *p* = 0.005) (Table 1).

There were not significant differences in IR, IS and pancreatic β cell function at 12 months between the study groups, and although both groups improved pancreatic β cell function, insulin secretion and pancreatic β cell function were improved slightly better in the II group (Table 1, *p* = 0.04). After 12 months of follow-up, a similar T2D incidence rate was observed in groups, 3.0 and 3.5% for II and FI group, respectively.

In family members of the II group, the slight and favorable changes achieved in several anthropometric characteristics like weight, waist circumference, fat percentage and visceral index at 6 months were lost at 12 months of follow-up and even worsened with respect to the initial values (*p* < 0.05, Table 3). Conversely, in the FI group, the family members continued to improve (Table 3), although without significant differences in the change from 0 to 12 months between groups.

After 12 months of follow-up glucose values during the OGTT remained similar between family members of both groups. Family members of the FI group had improvements in triglycerides, total cholesterol, HDL and LDL (Table 3), in contrast, lipid profile worsened in the II group; these improvements in total cholesterol (*p* = 0.01) and LDL (*p* = 0.003) where significantly better in the FI groups vs II group.

Finally, IS improved slightly in family members of the FI group at 12 months (without reaching significant difference, Table 3). Markers of insulin secretion and pancreatic β-cell function were not significantly different between groups.

## Discussion

Lifestyle interventions are effective to reduce T2D risk [25–27]. The challenge is how to convince the patient to improve and maintain the changes in their lifestyle.

Usually, patients with prediabetes face alone the treatment to control glucose levels. However, a key point to convince the patient to modify their lifestyle is the family support. Family could play a key role in the metabolic control of the patients, it could provide a favorable environment that reduces stress and improves compliance. In this work, patients with prediabetes faced the lifestyle intervention and pharmacologic treatment with their family members.

Of note, at 6 moths of follow-up, both groups of patients had significant improvements in almost all anthropometric characteristics without differences between groups (Table 1). II group achieved weight loss of 7% while FI group only lost 4%. Nevertheless, after 12 months of follow-up the landscape changed, since the improvements achieved after 6 months in waist circumference, body fat and visceral index in patients of II group were lost, returning to basal or even higher values (Table 1). Conversely, patients from the FI group continued losing weight at 12 months of follow-up (7%).

At 1 year of follow-up in the DPP study [7] the observed body weight loss was 2 and 6% in the metformin group and the intensive lifestyle group, respectively. A study conducted on Latin women reported a weight loss of 1 and 5% in the metformin group and in the intensive lifestyle intervention, respectively, after 1 year of follow-up [26]. In another study in which no medication was indicated, control group (general recommendations) lost 1% of weight and intervention group (intensive lifestyle modification), lost 4%, at 1 year of follow-up [6]. In the present work, at 1 year of follow-up, a weight loss of 6.2 and 7.2% was achieved in II and FI group, respectively.

Few studies have used metformin together with lifestyle modifications; the weight loss observed at 1 year of follow-up in these investigations was lower (− 4.2 [27] and − 2.5% [28]) than in the present study. The combined use of metformin, general assessment at every follow-up visit, adjustment of the diet every 2 months in II group, as well as the monthly sessions and the family support in the FI group, could be important factors that allowed a greater weight loss in our study groups.

### Lifestyle

The changes observed in calorie intake and physical activity were not significant in any of the intervention groups at 6 and 12 months of follow-up with respect to baseline values, or between groups. At 6 months of follow-up, the reduction in caloric intake in II group was 61 kcal and in FI group was 154 kcal. In the DPP study, after 1 year of follow-up, it was also reported a reduction in caloric intake in their intervention groups: 249 kcal in the placebo, 296 kcal in the metformin and 450 kcal in the intensive lifestyle change, with significant difference between groups.

In our study, it was observed that, after 6 months of follow-up, patients of FI group showed a greater reduction in caloric intake than patients of II group (− 7.6 and - 3.2% respectability), this could be due to the accompaniment of family members, since they also had to adhere to an established diet.

After 6 months of follow-up, patients in the FI group increased PA by 12.4% compared to patients in group II, which decreased 7.5%. At 12 months, PA decreased in both groups (− 15 in FI group and − 24.4% in II group). In contrast, at 1 year of follow-up, in the DPP study [7], the lifestyle group increased 7 times the PA. The difference could be because the participants of DPP study had 16 sessions the first 24 weeks, afterwards, the participants received individual and group sessions monthly to reinforce the changes in behavior on diet, exercise and behavior modification; the motivation and feedback was open and accessible to the participants, they had optional group courses quarterly with a duration of 4–6 weeks, and every 2 months the managers were personally in contact with the participants for the rest of the program. Our study was smaller and shorter, there were only 6 monthly group sessions and the multidisciplinary team of professionals had personal contact with the patient only once a month.

### Biochemistry characteristics

Patients in both groups had significant improvements in glucose levels at 6 months of follow-up and remained at 12 months, without significant changes between groups (Table 1). In contrast to other investigations, in our research we observed a greater decrease in fasting glucose values (− 7.0 and − 5.7%, II and FI group, respectively) than in those investigations in which only modification in the lifestyle was made (− 3.7%) [6]. Beside this, when comparing our results with fasting glucose reduction from other studies ((from − 1.9 to − 4.5%) in which they also used lifestyle modification plus metformin, we observed that our groups had greater fasting glucose reductions [27, 28].

The decrease in fasting glucose in the present study may be due to the general feedback and follow-up monthly of the group II, to the family support in the FI group and to the fact that both groups received metformin from the beginning of the intervention, unlike other studies in which metformin was not prescribed to all patients, and in some of them it was started after 4 months from the beginning of the intervention [27, 28].

After 6 months of follow-up, lipid profile in FI group had greater improvement and remained at 12 months of follow-up (Table 1). Tuomilehto J et al. (2001) [6] also reported improvement in their lifestyle group at 1 year of follow-up, their participants showed the following changes: − 5, + 2 and − 18 mg/dL in total cholesterol, HDL and triglycerides, respectively. In the present work, at 1 year of follow-up, the FI group had the following changes: − 11, + 2 and + 8 mg/dl in total cholesterol, HDL and triglycerides, respectively. The greater decrease at 1 year of follow-up in total cholesterol in our FI group is similar to that reported by Gokulakrishnan et al. (2016), who also used metformin plus lifestyle [27].

Furthermore, the magnitude of the improvement of the total cholesterol at 6 and 12 months and the LDL at 12 months of the FI group with respect to II group (*p* < 0.05) reflects the better control of the lipid profile in the FI group, which suggests that family support may have an important role on this aspect.

### Markers of insulin resistance and B cell function

Markers of IR and pancreatic β-cell function have an important role in the progression from prediabetes to T2D. Few studies focused on T2D prevention have measured glucose metabolism together with this markers, specifically pancreatic β-cell function, which seems to be the most important T2D predictor. Slentz CA et al. found that different programs based on exercise and nutrition improved significantly IS [29], similar to what we found in our study; however, O’Brien MJ et al. reported only modest improvements on IR in patients treated with metformin but not in patients included in a lifestyle program only [26]. Here, we found significant improvements on IR as well as in pancreatic β-cell function. No previous studies based on lifestyle programs have reported changes on pancreatic β-cell function.

### Family members

To our knowledge, no study has involved family in the treatment of patients with prediabetes. This is important because patients´ family members in our work had overweight, and this is a well-known risk factor for prediabetes or diabetes according to the ADA. At 1 year of follow-up, family intervention had a greater impact on BMI and anthropometrics characteristics than individual intervention and although family members of both groups did not have dyslipidemia, family intervention was also more effective in improving lipid profile. Therefore, it is possible to notice a better impact of interdisciplinary and family intervention, decreasing risk factors, which eventually will help to prevent prediabetes or diabetes.

### Markers of insulin resistance and B cell function

We found improvements on IS only in the family members of the FI group but not in the family members of the II group.

For all the above and due to the homogeneity presented by the groups at the beginning of the intervention, not only in the anthropometric and metabolic characteristics, but also in the socio-demographic characteristics and family history of T2D, we can indicate that the family interdisciplinary intervention had better impact than the individual one on the patients and the family members. The family support and the combination of the pharmacological treatment allowed greater improvement in weight, glucose and lipid profile than other interventions [4, 6, 7, 27, 28].

Although other researchers had already highlighted the importance of family support for glycemic control in T2D [11, 12], this is the first time that family members were included in a lifestyle intervention as a part of the treatment for patient with prediabetes. The interdisciplinary family intervention proposed here allowed patients to have the emotional support that facilitates changes in lifestyle, and at the same time, metabolic diseases can be prevented in the family members.

The family interdisciplinary strategy that was applied in the present investigation is viable and cost-effective compared to the expenses that are generated by the complications of diabetes; there were six monthly group sessions in which a group of specialists from different areas advised the participants on a better lifestyle and stress management, sessions of 1 h duration and monthly monitoring to review adherence to pharmacological treatment, diet and exercise, and recording of weight and glucose.

It will be necessary to follow up these patients and their families for a long term because it has been shown in other investigations [4, 8] that the improvements that are achieved in the first years can partially be reduced or even lost after the finishing of the study. In addition, it will also be necessary to analyze the best strategy to give feedback to the participants and their families to maintain the achieved changes for longer time.

### Study limitations

The study has several limitations: we did not have the complete data on the change in caloric intake and physical activity of the participants of both groups at 6 and 12 months, therefore, it was not possible to have a real landscape of these aspects and their possible impact on the main outcomes; future research will be necessary with a better control of these aspects. Another limitation of our study was the high percentage of dropouts, which reflects the reality and the lack of availability of the Mexican population with prediabetes to invest time in health care and disease prevention. It is evident that the work must continue in favor of prevention, biggest and largest studies focusing on family interventions would be needed in different populations to encourage changes in public policies to include family members in the treatment and prevention of cardiometabolic diseases.

Although there was a high percentage of drop-outs, we believe that our results are valid and contribute to this research area, considering the difficulties in the longitudinal follow-up in group interventions, and besides that, participants who finished the 12-month follow-up had similar glucose metabolism, anthropometric and pathophysiological characteristics to those who didn’t continue the follow-up.

## Conclusions

The family interdisciplinary intervention, including the support of family members, was more effective in maintaining the improvements of glucose metabolism, IR, lipid profile and body composition. In addition to this, family members of the FI group maintained their metabolic profile and body composition after 12 months of follow-up. This kind of preventive strategies could be cost-effective at high scale, but more studies are needed to confirm this.

## Supplementary information

**Additional file 1.** Interdisciplinary Family Intervention. Detailed description of the interdisciplinary family intervention

## Data Availability

The datasets used and/or analyzed during the current study are available from the corresponding author on reasonable request.

## Abbreviations

ADA: American Diabetes Association
AU: Arbitrary units
AUC: Area under the glucose curve
AUCgluc_OGTT: Area under the glucose curve during oral glucose tolerance test
AUCIns: Area under the insulin curve.
BMI: Body mass index
CARE-in-DEEP: Cardiometabolic Risk Evaluation and Interdisciplinary Diabetes Education and Early Prevention
DPP: Diabetes Prevention Program
FI: Family intervention
FPG: Fasting glucose
HbA1c: Glycated hemoglobin
HDL: High density lipoprotein
HOMA-IR: Homeostatic model assessment-IR.
II: Individual intervention
IR: Insulin resistance
IS: Insulin sensitivity
LDL: Low density lipoprotein
OGTT: Oral glucose tolerance test
PA: Physical activity
RCT: Randomized Clinical Trial
SBP: Systolic pression blood
T2D: Type 2 diabetes
Tg: Triglycerides
WC: Waist circumference
