# Supplementary material for: Effect of a family and interdisciplinary intervention to prevent T2D: randomized clinical trial
Source: BMC Public Health. 2020 Jan 22;20:97. doi: 10.1186/s12889-020-8203-1 (PMC6977289; doi:10.1186/s12889-020-8203-1)
Supplement: Supplementary file 1 — Additional file 1. Interdisciplinary Family Intervention. Detailed description of the interdisciplinary family intervention [file 12889_2020_8203_MOESM1_ESM.docx]

Additional file 1.

**Interdisciplinary Family Intervention**

Like the patients of the individual intervention, the patients of the interdisciplinary family intervention ingested 850 mg of metformin twice daily, they received a diet consisting of 50 - 60% carbohydrates, 15 - 20% proteins and less than 30% total fat. In case of overweight or obesity the patients received detailed nutrition advice to achieve a 5-7% reduction in body weight and a moderate caloric restriction to lose weight was prescribe (250 to 500 kilocalories less than ingestion daily average calculated in the dietary regimen). Dietary recommendations were customized considering the 3-day food records; the decrease in caloric intake was gradual through the months, adjusting the diet every two months. If the patient was sedentary, it was advised to start with 45 min/week of mild to moderate exercise (the chosen activity was according to patient preference), a daily frequency was recommended or at least every third day. Patients were counseled to increase the time or intensity of exercise every two weeks until reaching 150 min/week of moderate activity or 75 min/week of intense activity. If the patient was already physically active, it was recommended to continue like this and vary its exercise routines.

The patients had monthly follow-up for 12 months. At each follow-up visit the body composition, fasting glucose, adherence to pharmacotherapy (by pill count), diet and exercise were revised (by 3-day food records and the International Physical Activity Questionnaire [IPAQ]). The pertinent recommendations were given to each patient in order to achieve better adherence in all aspects of the intervention.

Patients and their family members attended a monthly lifestyle enhancement program.

**Group sessions**

There were 6 sessions, one session every month during the first 6 months with an approximate duration of 1h. For session 1, only 3 families were attended to explain the food plan individually, the rest of the sessions were attended between 4 - 6 families. The next table describe the topics of each session by discipline.

| Session number | Discipline | Topics |
| --- | --- | --- |
| 1 | **Psychology** | Presentation of topics to be reviewed during the sessions. |
|  | **Nutrition** | Delivery, interpretation and use of individualized food plan. |
|  | **Physical Activity** | Presentation of topics to be reviewed during the sessions. |
| 2 | **Psychology** | Family support.  Tips to achieve changes: Exercises about personal commitment, self-image and sensations / Self-esteem. |
|  | **Nutrition** | Plate of good eating / Characteristics of a proper diet. |
|  | **Physical Activity** | General guidelines about quantity and correct way of exercising.  Tips to increase physical activity throughout the day.  Advices for a sedentary subject to start exercising. |
| 3 | **Psychology** | Relationship between body mind and emotions.  Mindfulness about thinking.  Tips for quieting the mind: Positive attitude / Practice the thanks /  To meditate. |
|  | **Nutrition** | Answers to questions about the individualized food plan.  Food groups. |
|  | **Physical Activity** | Benefits of the exercise.  Performing physical activity safely. |
| 4 | **Psychology** | Review of adherence to the commitments established at the beginning of the program.  How to use a commitment logbook and its benefits.  Practice a relaxation exercise: body scan. |
|  | **Nutrition** | How to interpret food product labels:  Serving size and number of servings per package.  Calories per serving and per package.  Identify the nutrient present in greater quantity.  Check amount of sugar, dietary fiber, fat and sodium. |
|  | **Physical Activity** | Types of exercise and its benefits.  Combination of types of exercise during the week or during a session. |
| 5 | **Psychology** | Relaxation exercise practice: Visualization exercises. |
|  | **Nutrition** | Food supplements: Definition, use and Benefits and risks. |
|  | **Physical Activity** | Physical activity at home: How to select an exercise video / Appropriate and safe routines / Demonstration and practice of a routine to strengthen the main muscle groups. |
| 6 | **Psychology** | Five pillars of well-being:  1 Positive emotions / 2 Personal commitment / 3 Process relations / 4 Search for activities that make sense of life / 5 Have goals and achievements. |
|  | **Nutrition** | Myths and realities of nutrition: Vitamin intake / Bread consumption / Water amount / Light food products / Prohibited foods for diabetics / Single-food diets / Alcoholic drinks. |
|  | **Physical Activity** | Sedentary and detraining / Sedentary risks.  Progression of exercise routines. |
